# Increased bcl-2 Protein Levels in Rat Primary Astrocyte Culture Following Chronic Lithium Treatment

**Published:** 2013-09

**Authors:** Mojtaba Keshavarz, Masoumeh Emamghoreishi, Ali Akbar Nekooeian, Jerry J. Warsh, Hamid Reza Zare

**Affiliations:** 1 Department of Pharmacology, School of Medicine, Shiraz University of Medical Sciences, Shiraz, Iran;; 2 Laboratory of Cellular and Molecular Pathophysiology, Centre for Addiction and Mental Health, and Department of Psychiatry, Pharmacology and Toxicology, and Institute of Medical Science, University of Toronto, Toronto, Ontario, Canada;; 3 Department of Immunology, School of Medicine, Shiraz University of Medical Sciences, Shiraz, Iran

**Keywords:** Lithium, bcl-2, Astrocytes, Primary cell culture, Neuron

## Abstract

**Background:** B cell CLL/lymphoma 2 protein, bcl-2, is an important anti-apoptotic factor that has been implicated in lithium’s neuroprotective effect. However, most studies have focused on assessing the effects of lithium in neurons, ignoring examination of bcl-2 in astrocytes, which also influence neuronal survival and are affected in bipolar disorder. The aim of this study was to evaluate whether chronic lithium treatment also elevates bcl-2 expression in astrocytes compared with neuronal and mixed neuron-astrocyte cultures.

**Methods:** Rat primary astrocyte, neuronal, and mixed neuron-astrocyte cultures were prepared from the cerebral cortices of 18-day embryos. The cell cultures were treated with lithium (1 mM) or vehicle for 24 h or 7 days. Thereafter, bcl-2 mRNA and protein levels were determined by RT-PCR and ELISA, respectively.

**Results: **Chronic, but not acute, lithium treatment significantly increased bcl-2 protein levels in the astrocyte cultures compared with the vehicle-treated cultures. While lithium treatment increased bcl-2 protein levels in both neuronal and mixed neuron-astrocyte cultures, the elevations fell short of statistical significance compared with the respective vehicle-treated cultures. However, neither acute nor chronic lithium treatment affected bcl-2 mRNA levels in any of the three cell types studied.

**Conclusion:** Increased bcl-2 levels in rat primary astrocyte cultures following chronic lithium treatment suggest astrocytes are also a target of lithium’s action. In light of the evidence showing decreased numbers of glial cells in the post-mortem brain of patients bipolar disorder with and increased glial numbers following lithium treatment, the findings of this study indicate that lithium’s action on astrocytes may account, at least in part, for its therapeutic effects in bipolar disorder.

## Introduction

Although lithium has been used for a long time as an accepted pharmacological treatment for bipolar disorder (BD), its mechanism of action is not yet precisely clear. Substantial evidence indicates that intracellular signaling systems involved in neuroprotection are an important target for lithium’s mood stabilizing and neuroprotective effects.^1^ In this regard, B Cell CLL/lymphoma-2 protein (bcl-2), which is an anti-apoptotic member of the bcl-2 protein family, has been implicated as a key player in the neuroprotective actions of lithium^2^ and the pathophysiology of BD.^3^ Several lines of evidence support the association between bcl-2 in the pathophysiology of BD and the mechanism of action of mood-stabilizing agents.^4^ An association between bcl-2 and manic-like behavior has been demonstrated using bcl-2 deficient mice.^5^ Moreover, a bcl-2 polymorphic intronic variant has been found to be allied to reduced ventral striatum gray matter volume.^6^ Reduced cortex grey matter volume has been reported in post-mortem brain^7^ and structural neuroimaging studies of BD.^8^ Notably, lithium treatment has been reported to increase gray matter volume in bipolar patients^9^ and to enhance the expression of bcl-2 in rat brain.^10^ These findings, together with animal and cellular studies of the effects of mood stabilizer on bcl-2,^11^ have led to the notion that the upregulation of bcl-2 levels in brain may mediate, in part, the neuroprotective effect of lithium.^11^

Almost all of the studies investigating the mechanism of action of lithium have focused on neurons as its primary target. However, there is growing evidence implicating a role for glial cells in the process of neuroprotection.^12^ In this regard, astrocytes play significant roles in regular neuronal action by regulating extracellular ions and neurotransmitters and by making available energy substrates.^13^ In addition, some studies have shown that the over-expression of bcl-2 in astrocytes increases neuronal survival against stressors, an effect that is attributed to enhanced astrocyte function during stress.^14^ In agreement with this idea, it has been demonstrated that the sensitivity of neurons to stressors (e.g. glutamate toxicity) is significantly lower in astrocyte-rich than in astrocyte-poor cultures.^15^ These findings indicate that the impaired function or loss of astrocytes can lead to neuronal death or dysfunction.^15^ This, together with the evidence of decreased numbers of glial cells in post-mortem BD brain^16^ and the apparent effect of lithium to prevent such changes,^17^ led us to propose that lithium may act indirectly to improve the function of neurons by protecting astrocytes from apoptosis via increasing bcl-2 levels, in addition to direct effects on neuronal bcl-2 expression. Therefore, the main objective of this study was to determine the effects of lithium on bcl-2 mRNA and protein levels in rat primary astrocyte cultures in contrast to its effects on bcl-2 in neuron and mixed neuron-astrocyte cultures.

## Materials and Methods

*Chemicals and Reagents*

Neurobasal media, Dulbecco's Modified Eagle's Medium (DMEM), B27 supplement, heat-inactivated horse serum (HS), G_5_ supplement, and trypsin–ethylene-diamine-tetra-acetic acid (EDTA) (0.05%) were purchased from Gibco (USA). Rabbit polyclonal antibody to microtubule-associated protein 2 (MAP-2) and mouse monoclonal antibody [GF5] to glial fibrillary acidic protein (GFAP) were obtained from Abcam (USA). Cytosine arabinoside (ara-c), polyethylene imine (PEI), leucine-leucine methyl ester, diamidinophenylindole (DAPI), and NP40 were purchased from Sigma (USA). Hank’s Balances Salt solution (HBSS), penicillin-streptomycin, and l-glutamine were provided from BioSera (England). Other reagents were obtained as follows: lithium chloride (Merck, Germany); TriPure Isolation reagent (Roche, USA); revertaid H minus first strand cDNA synthesis kit (Fermentas Life Science, USA); SYBR green I kit (ABI, Singapore); bcl-2 ELISA kits (BlueGene, China); Alexa Fluor 594 goat anti-rabbit (Invitrogen); Alexa Fluor 488 goat anti-mouse (Invitrogen); and Image-iT FX Signal Enhancer (Invitrogen).

*Fetal Rat Cortex Dissection*

Embryonic cortices were obtained from 18-day embryos of Sprague-Dawley rats (N=7) using a modification of the method of Cole et al.^18^ The rats were handled according to the guidelines for animal care and with the approval of the Ethics Committee of Shiraz University of Medical Sciences. 

The cortices were dissected and triturated with a fire-polished Pasteur pipette in cold HBSS, followed by centrifugation at 800 x g for 10min. Precipitated cells were re-suspended in HBSS and used for different primary cultures–as is mentioned below. Viable cells were counted using phase-contrast microscopy (Micros, Austria) and Trypan Blue. 

*Preparation of Rat Primary Neuronal Cultures*

The cells (3.5×10^6^) were seeded in 60 mm PEI-coated dishes in neurobasal media supplemented with 10% HS, 2 mM l-glutamine, 50 unit/ml penicillin, and 50 µg/ml streptomycin. The cultures were kept at 37°C in a 5% CO_2_, 95% O_2_ humidified incubator. After 24h, HS in the neurobasal medium was replaced with 2% B27. Seventy-two hours after plating, ara-c (10 µM final concentration) was added for 24h to prevent non-neuronal cell proliferation. The neuronal cultures were exposed to lithium (1 mM final concentration) or vehicle (distilled sterile water) either for 24 h (acute) or 7 days (chronic) starting on day 7 of culturing. The media were replenished every other day during the 7 days of lithium exposure. 

*Preparation of Rat Primary Astrocyte Cultures*

The cells (10-20×10^6^) were transferred to 175-cm^2^ un-coated culture flasks in DMEM containing HS (10%), l-glutamine (2 mM), penicillin (50 U/ml), and streptomycin (50 µg/ml). The medium was changed 72h after the initial seeding, and then on alternate days. Upon reaching confluence (~3 weeks), astrocytes were separated using standard shaking procedures.^19^ After 72h, the purified astrocytes were detached by trypsin–EDTA (0.05%) and seeded in the 10 cm PEI-coated dishes, containing the same culture medium. When the cells reached confluence, HS was replaced with 1% G5 as a serum-free supplement, and the cultures were exposed to lithium (1 mM) or vehicle (distilled sterile water) for 24h or 7 days.

*Rat Primary Mixed Neuro-Astrocyte Cultures*

Neuron-astrocyte cultures were prepared following the method of Hong and La.^20^ In brief, dissociated cells (5×10^6^) were seeded in PEI-coated dishes in DMEM medium with 10% HS, 2 mM l-glutamine, 50 U/ml penicillin, and 50 µg/ml streptomycin and subsequently kept under the same above-mentioned conditions (section 2.3). After 72h, B27 (1%) was added to the culture medium. On the 4^th^ day, 1.5 mM leucine-leucine methyl ester was added to the medium to deplete microglia from neuron-glia mixed cultures. On the 8^th^ day, HS was replaced with 1% G5 supplement, and the cells were exposed to lithium (1 mM) or vehicle (distilled sterile water) for 24h or 7 days. 

*Immunocytochemistry*

Purity of the cell cultures was confirmed via immunocytochemistry, as was described by Chamak et al. (1987) with modifications.^21^^,^^22^ After removing the media, the cells were fixed in 4% formaldehyde for 15min at 37°C, followed by incubation with blocking solution for 1h at room temperature (R.T.). The neurons and astrocytes were then incubated with MAP-2 (1:100 dilution) and GFAP antibodies (1:100 dilution), respectively, at RT for 2h. After washing, the cells were exposed to secondary antibodies at 1:400 dilutions (Alexa flour 596 for MAP-2 and Alexa flour 488 for GFAP) and incubated for 1.5h at R.T. Finally, the cell nuclei were counterstained with DAPI. The cells were visualized and counted via fluorescence microscopy (Canon, Japan) at a magnification of 100x in four representative areas per cover slip. 

*Quantitative RT-PCR for bcl-2*

Total RNA was extracted via the phenol-chloroform extraction method using TriPure Isolation reagent in accordance with the manufacturer’s instruction.^23^ cDNA was synthesized from 1μg total RNA using the revertaid H minus first strand cDNA synthesis kit according to the manufacturer’s guidelines. The relative levels of bcl-2 [RefSeq: NM016993] and GAPDH [RefSeq: NM017008.3] mRNAs were determined using quantitative real time PCR (RT-PCR) using an ABI PRISM 7500 real-time PCR system (Applied Biosystem, USA). Specific primers were: for bcl-2, forward primer 5-CCT GCC CCA AACAAA TAT GAA AAG-3 and reverse primer 5- TTG ACC ATT TGCCTG AAT GTG TG-3; and for GAPDH, forward primer 5- CGT GAT CGAGGGCTGTTG G-3 and reverse primer 5-CTGCTTCAGTTG GCC TTT CG-3. The primers were designed to span exon-exon junctions in order to preclude the amplification of genomic DNA. The targets amplicon sizes were 174bp and 97bp for bcl-2 and GAPDH, respectively. The RT-PCR reaction condition was as follows: initial denaturation at 94°C for 3min, followed by 35 cycles of denaturation at 94°C for 30sec; annealing at 58°C for 30sec; and extension at 72°C for 45sec. The threshold cycles (Ct) of the samples were used to calculate the ratio of expressions between the lithium-treated and untreated samples. 

*ELISA for Quantification of bcl-2 Protein Levels*

The cells were lysed by NP40 buffer and stored at -70°C until assay. Total protein was measured via the Bradford method^24^ using 6 concentrations of BSA as standards. Additionally, bcl-2 protein levels were quantified using a Bluegene rat bcl-2 ELISA kit, which contained a highly specific bcl-2 antibody with no significant cross-reactivity with other bcl-2 analogues. Briefly, cell lysates were added to the wells, pre-coated with polyclonal anti-bcl-2 antibody and a bcl-2-HRP conjugate, and incubated for one hour at 37^o^C. The wells were washed and incubated with tetramethylbenzidine as the HRP substrate at R.T. for 15 min. After adding stop solution, the absorbance was measured at 450nm in a micro-plate reader (Micura, England). The bcl-2 concentrations were interpolated from the standard curves using samples with known bcl-2 concentrations (25-500 pg/ml). The relationship between total protein concentration (20-500 mg) and absorbance intensity was best fit by a quadratic function (y=0.868x^2^-1.899x+1.062, R²=0.951), which was used to estimate bcl-2 immunoreactivity levels. The intra-assay and inter-assay coefficient of variance was 5% and 10%, respectively.

*Statistical Analysis*

The data are expressed as mean±SEM for each group. Due to the different amplification efficiency of bcl-2 and GAPDH, the Pfaffl method of REST software (REST-384-beta)^25^ was employed to compare bcl-2 mRNA expression levels between the lithium and vehicle-treated cells. Differences in bcl-2 protein levels between the lithium and vehicle-treated cells were assessed using paired *t-*test. The relative changes of bcl-2 levels in the lithium-treated cultures, expressed as a percent of the vehicle-treated cultures, were compared between the three cell types cultured using one-way ANOVA and post hoc comparisons with the LSD test. SPSS software (version 11.5) was used for the statistical analyses. A P value≤0.05 was considered statistically significant. 

## Results

*Immunocytochemistry*

**Figure 1:**
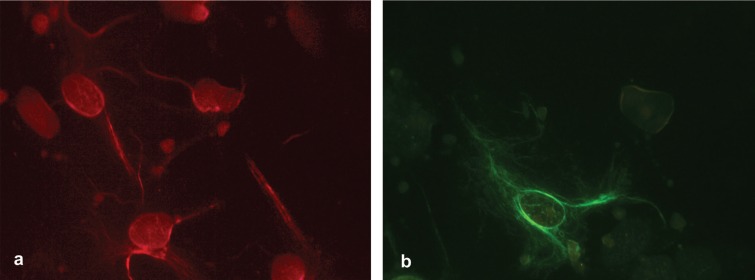
Immunocytochemical staining of a) neuronal cultures with anti-MAP2 antibody and b) astrocyte cultures with anti-GFAP antibody indicated neurons (red fluorescence) and astrocytes (green fluorescence) purities of greater than 90%, respectively. MAP: microtubule-associated protein 2; GFAP: glial fibrillary acidic protein

Primary cultures were successfully grown from cell suspension of fetal rat cortices. The immunocytochemical staining positively identified the neurons (glow red fluorescence) and astrocytes (glow green fluorescence), growing in the cultures (figure 1). The results of immunofluorescence showed that the neuronal and astrocyte cultures were enriched, containing more than 90% neurons (figure 1a) and astrocytes (figure 1b), respectively. The mixed neuron-astrocyte cultures were comprised of approximately 55% astrocytes and 44% neurons. 

*Effects of Chronic Lithium Treatment on bcl-2 mRNA and Protein Levels*

**Figure 2:**
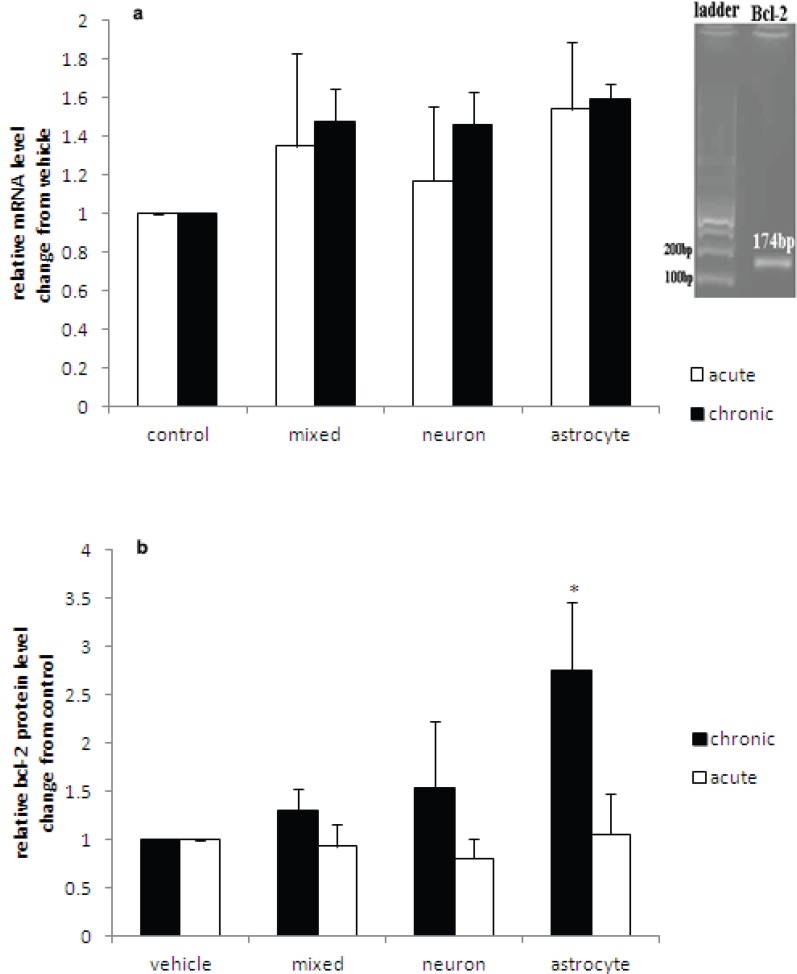
Effects of acute and chronic lithium treatment on bcl-2 a) mRNA and b) protein levels in rat primary astrocyte, neuronal, and mixed astrocyte-neuronal cultures treated with lithium (1 mM) or vehicle (100 µl sterile distilled water, control) for one day (acute) or 7 days (chronic). Bars indicate the mean±SEM, n=7 independently grown and treated cell lines per group. *indicates P<0.05 compared to vehicle-treated cultures

Quantitative real-time PCR was performed to evaluate the effect of lithium treatment on bcl-2 mRNA levels. Gel electrophoresis of the PCR products showed the specific amplification of a single 174bp amplicon, as was expected for the target (figure 2a). Lithium increased bcl-2 mRNA levels in the rat primary astrocyte, neuron, and mixed neuron-astrocyte cultures by factors of 1.59±0.08, 1.46±0.17, and 1.48±0.17, respectively, in comparison to their respective vehicle-treated cultures (figure 2a). However, these changes were not statistically significant (P=0.33, 0.64, and 0.57, respectively). In addition, there were no significant differences in the fold increases in bcl-2 mRNA levels of the lithium-treated cultures as compared with the vehicle-treated cultures between the three different cultures.

Chronic treatment with lithium increased bcl-2 protein levels significantly in the astrocyte (175%; P<0.05) (figure 2b), but not in the neuronal (53%) or mixed neuron-astrocyte (30%) cultures in comparison to their respective vehicle-treated cultures. There were no significant differences in the basal level of bcl-2 protein (in the absence of lithium) (F=0.49, df=2; P=0.62) or percent change from basal bcl-2 protein level (F=1.66, df=2; P=0.22) between the neuronal, astrocyte, and mixed cultures. Moreover, acute lithium treatment (1 mM for 24 h) had no significant effects on bcl-2 protein levels in the mixed neuron-astrocyte, neuron, and astrocyte cultures in comparison to the respective vehicle-treated cultures (P=0.89, 0.50, and 0.89, respectively) (figure 2b).

## Discussion

The objective of the present study was to investigate the effects of chronic lithium treatment on bcl-2 levels in three different cell type preparations enriched from rat primary cortical cultures. The findings indicate that chronic, but not acute, lithium treatment increased bcl-2 protein levels in the rat primary astrocyte cultures and exerted a trend increase in the neuronal culture. In contrast, neither acute nor chronic lithium treatment affected bcl-2 levels in the mixed neuron-astrocyte culture. To our knowledge, this study is the first of its kind to report that 7 days of lithium treatment (1 mM) increases bcl-2 protein levels in rat primary cortical astrocyte cultures. Although several earlier studies examined bcl-2 expression following chronic lithium exposure, those works were performed on transformed glial cell models, including human SVG p12 astroglial cells, rat C6 glioma cells,^26^ and U87 human glioma cells, with and without ER stress induction,^27^ no changes were found with lithium at therapeutically relevant concentrations (0.75-2 mM). The effect of immortalization of the studied cell types may have obscured the induction of bcl-2 expression changes by lithium. The effect of lithium treatment of primary astrocytes derived from rat cerebral cortices, as was the case in this study, may have more resemblance to the effect that it actually exerts in astroglial cells in vivo. Moreover, our findings support the notion that astroglial cells also may be an important target of lithium action in brain.

In this study, lithium increased the protein, but not mRNA, levels of bcl-2 in astrocytes. This non-correspondence between changes in mRNA and protein levels has been previously reported for lithium’s effects on BDNF in the rat hippocampus and frontal cortex.^28^ This increase in bcl-2 protein levels without an associated change in mRNA levels may reflect either post-transcriptional alteration that decreases mRNA stability^29^ or a post-translational modification that reduces the rate of bcl-2 degradation. This notion is supported by an earlier finding that lithium inhibited proteasomal degradation, leading to increased levels of some proteins without altering their respective mRNA levels, as was shown in keratinocyte cell lines.^30^

The lack of a statistically significant increase in bcl-2 mRNA or protein levels in primary neuronal and mixed neuron-astrocyte cultures following lithium treatment agrees with those findings of a previous report that a one-week treatment of human hNT neurons with lithium (0.75-2 mM) did not change bcl-2 mRNA levels.^26^ Moreover, they concur with those of an *in vivo *study in which 14 days of treatment with therapeutic doses of lithium did not affect bcl-2 levels in the dendate gyrus and area CA1 of adult rat hippocampus.^31^ On the other hand, the present findings are not consistent with previously published reports that chronic lithium treatment in certain neuronal cell models or in vivo increased bcl-2 protein or mRNA levels.^2^^,^^27^ Such discordance might be due to cell type dependent differences, cell culture conditions, region of brain studied, duration and concentration of lithium treatment, experimental conditions such as the use of stressed versus unstressed cells, or experimental designs (i.e. in vivo versus in vitro studies). For instance, some studies used neuronal cell lines of non-CNS origin such as SH-SY5Y or PC12 cells,^27^^,^^32^ or used neurons from cerebellum,^33^ for which there is little evidence to support its involvement in BD. It is also plausible that lithium may regulate the interplay between various cells in brain cellular matrix, affecting neuronal bcl-2 in vivo^2^^, ^^34^ differently than in a reconstituted mixture of neurons and astrocytes in vitro. 

The basis for the finding that chronic lithium treatment increased bcl-2 protein levels in the astrocytes but not in the mixed neuron-astrocyte culture is not completely clear. It is possible that lithium does not affect the interaction between neurons and astrocytes in vitro under non-stressed conditions. Song et al.^35^ reported earlier that lithium exerted some of its effects only when cells were stressed. Further studies are necessary to evaluate the effect of lithium on bcl-2 in rat primary neuron-astrocyte cultures in the presence and absence of stressors. 

Elevation of bcl-2 levels in cortical astrocytes by lithium may have important implications with respect to its spectrum of therapeutic actions. Decreased gray matter volume, especially in prefrontal cortex,^36^ may be at least partly attributed to the decreased number and/or size of astrocytes.^16^^,^^37^ Astrocytes constitute the majority of glial cells in the CNS;^16^ reduced glial density has been shown in BD post-mortem cortical brain regions.^38^ Taken together, structural neuroimaging and post-mortem brain findings implicate astroglial impairment in BD and lend support to the notion that the efficacy of lithium in the treatment of BD may be partly mediated by enhancing astroglial integrity. Such a conclusion is also supported by earlier studies showing that lithium treatment can increase gray matter volume in BD brains, that mood stabilizers appear to prevent glial reduction in BD brain,^9^^,^^17^ and that lithium protected astrocytes from apoptosis by inhibiting glycogen synthase kinase-3β inhibition,^39^ which elevates bcl-2 levels.^40^ Thus, it is plausible that the effect of lithium to increase the level of bcl-2 in astrocytes is also important to this glial-targeted effect. 

## Conclusion

**C**hronic lithium treatment increased bcl-2 protein levels in the primary cortical astrocytes but not in the neuronal cultures. These findings highlight astrocytes as a target of chronic lithium action, which may be relevant to its therapeutic effects in BD. 

## References

[1] Chuang DM (2005). The antiapoptotic actions of mood stabilizers: molecular mechanisms and therapeutic potentials. Ann N Y Acad Sci.

[2] Chen G, Zeng WZ, Yuan PX, Huang LD, Jiang YM, Zhao ZH (1999). The mood-stabilizing agents lithium and valproate robustly increase the levels of the neuroprotective protein bcl-2 in the CNS. J Neurochem.

[3] Kim HW, Rapoport SI, Rao JS (2010). Altered expression of apoptotic factors and synaptic markers in postmortem brain from bipolar disorder patients. Neurobiol Dis.

[4] Manji HK, Chen G (2002). PKC, MAP kinases and the bcl-2 family of proteins as long-term targets for mood stabilizers. Mol Psychiatry.

[5] Lien R, Flaisher-Grinberg S, Cleary C, Hejny M, Einat H (2008). Behavioral effects of Bcl-2 deficiency: implications for affective disorders. Pharmacol Rep.

[6] Salvadore G, Nugent AC, Chen G, Akula N, Yuan P, Cannon DM (2009). Bcl-2 polymorphism influences gray matter volume in the ventral striatum in healthy humans. Biol Psychiatry.

[7] Drevets WC, Price JL, Furey ML (2008). Brain structural and functional abnormalities in mood disorders: implications for neurocircuitry models of depression. Brain Struct Funct.

[8] Savitz J, Drevets WC (2009). Bipolar and major depressive disorder: neuroimaging the developmental-degenerative divide. Neurosci Biobehav Rev.

[9] Lyoo IK, Dager SR, Kim JE, Yoon SJ, Friedman SD, Dunner DL (2010). Lithium-induced gray matter volume increase as a neural correlate of treatment response in bipolar disorder: a longitudinal brain imaging study. Neuropsychopharmacology.

[10] Chang YC, Rapoport SI, Rao JS (2009). Chronic administration of mood stabilizers upregulates BDNF and bcl-2 expression levels in rat frontal cortex. Neurochem Res.

[11] Chen G, Masana MI, Manji HK (2000). Lithium regulates PKC-mediated intracellular cross-talk and gene expression in the CNS in vivo. Bipolar Disord.

[12] Trendelenburg G, Dirnagl U (2005). Neuroprotective role of astrocytes in cerebral ischemia: focus on ischemic preconditioning. Glia.

[13] Pellerin L, Magistretti PJ (2003). How to balance the brain energy budget while spending glucose differently. J Physiol.

[14] Xu L, Lee JE, Giffard RG (1999). Overexpression of bcl-2, bcl-XL or hsp70 in murine cortical astrocytes reduces injury of co-cultured neurons. Neurosci Lett.

[15] Rosenberg PA, Aizenman E (1989). Hundred-fold increase in neuronal vulnerability to glutamate toxicity in astrocyte-poor cultures of rat cerebral cortex. Neurosci Lett.

[16] Ongür D, Drevets WC, Price JL (1998). Glial reduction in the subgenual prefrontal cortex in mood disorders. Proc Natl Acad Sci U S A.

[17] Bowley MP, Drevets WC, Ongür D, Price JL (2002). Low glial numbers in the amygdala in major depressive disorder. Biol Psychiatry.

[18] Cole R, Vellis J, Fedoroff S, Richardson A (2001). Preparation of Astrocyte, Oligodendrocyte, and Microglia Cultures from Primary Rat Cerebral Cultures. Protocols for Neural Cell Culture.

[19] Feeney CJ, Frantseva MV, Carlen PL, Pennefather PS, Shulyakova N, Shniffer C (2008). Vulnerability of glial cells to hydrogen peroxide in cultured hippocampal slices. Brain Res.

[20] Liu B, Hong JS (2003). Primary rat mesencephalic neuron-glia, neuron-enriched, microglia-enriched, and astroglia-enriched cultures. Methods Mol Med.

[21] Chamak B, Fellous A, Glowinski J, Prochiantz A (1987). MAP2 expression and neuritic outgrowth and branching are coregulated through region-specific neuro-astroglial interactions. J Neurosci.

[22] McLendon RE, Bigner DD (1994). Immunohistochemistry of the glial fibrillary acidic protein: basic and applied considerations. Brain Pathol.

[23] Chomczynski P, Sacchi N (1987). Single-step method of RNA isolation by acid guanidinium thiocyanate-phenol-chloroform extraction. Anal Biochem.

[24] Kruger NJ, Walker JM (2002). The Bradford Method for Protein Quantitation. The Protein Protocols Handbook.

[25] Pfaffl MW, Horgan GW, Dempfle L (2002). Relative expression software tool (REST) for group-wise comparison and statistical analysis of relative expression results in real-time PCR. Nucleic Acids Res.

[26] Corson TW, Woo KK, Li PP, Warsh JJ (2004). Cell-type specific regulation of calreticulin and Bcl-2 expression by mood stabilizer drugs. Eur Neuropsychopharmacol.

[27] Lai JS, Zhao C, Warsh JJ, Li PP (2006). Cytoprotection by lithium and valproate varies between cell types and cellular stresses. Eur J Pharmacol.

[28] Jacobsen JP, Mørk A (2004). The effect of escitalopram, desipramine, electroconvulsive seizures and lithium on brain-derived neurotrophic factor mRNA and protein expression in the rat brain and the correlation to 5-HT and 5-HIAA levels. Brain Res.

[29] Atwater JA, Wisdom R, Verma IM (1990). Regulated mRNA stability. Annu Rev Genet.

[30] Shim M, Smart RC (2003). Lithium stabilizes the CCAAT/enhancer-binding protein alpha (C/EBPalpha) through a glycogen synthase kinase 3 (GSK3)-independent pathway involving direct inhibition of proteasomal activity. J Biol Chem.

[31] Hammonds MD, Shim SS, Feng P, Calabrese JR (2007). Effects of subchronic lithium treatment on levels of BDNF, Bcl-2 and phospho-CREB in the rat hippocampus. Basic Clin Pharmacol Toxicol.

[32] Hiroi T, Wei H, Hough C, Leeds P, Chuang DM (2005). Protracted lithium treatment protects against the ER stress elicited by thapsigargin in rat PC12 cells: roles of intracellular calcium, GRP78 and Bcl-2. Pharmacogenomics J.

[33] Chen RW, Chuang DM (1999). Long term lithium treatment suppresses p53 and Bax expression but increases Bcl-2 expression A prominent role in neuroprotection against excitotoxicity. J Biol Chem.

[34] Chen G, Rajkowska G, Du F, Seraji-Bozorgzad N, Manji HK (2000). Enhancement of hippocampal neurogenesis by lithium. J Neurochem.

[35] Song N, Boku S, Nakagawa S, Kato A, Toda H, Takamura N (2012). Mood stabilizers commonly restore staurosporine-induced increase of p53 expression and following decrease of Bcl-2 expression in SH-SY5Y cells. Prog Neuropsychopharmacol Biol Psychiatry.

[36] Adler CM, Levine AD, DelBello MP, Strakowski SM (2005). Changes in gray matter volume in patients with bipolar disorder. Biol Psychiatry.

[37] Hajek T, Carrey N, Alda M (2005). Neuroanatomical abnormalities as risk factors for bipolar disorder. Bipolar Disord.

[38] Rajkowska G, Halaris A, Selemon LD (2001). Reductions in neuronal and glial density characterize the dorsolateral prefrontal cortex in bipolar disorder. Biol Psychiatry.

[39] Sanchez JF, Sniderhan LF, Williamson AL, Fan S, Chakraborty-Sett S, Maggirwar SB (2003). Glycogen synthase kinase 3beta-mediated apoptosis of primary cortical astrocytes involves inhibition of nuclear factor kappaB signaling. Mol Cell Biol.

[40] Manji HK, Moore GJ, Chen G (2000). Lithium up-regulates the cytoprotective protein Bcl-2 in the CNS in vivo: a role for neurotrophic and neuroprotective effects in manic depressive illness. J Clin Psychiatry.

